# Assessing the Consultation and Relational Empathy (CARE) Measure in sexual health nurses’ consultations

**DOI:** 10.1186/s12912-017-0265-8

**Published:** 2017-11-25

**Authors:** Annemieke P. Bikker, Bridie Fitzpatrick, Douglas Murphy, Lorraine Forster, Stewart W. Mercer

**Affiliations:** 1 Usher Institute for Population Sciences and Informatics, The University of Edinburgh, 9 BioQuarter, Little France Road, Edinburgh, EH16 4UX UK; 2 General Practice and Primary Care, Institute of Health and Wellbeing, University of Glasgow, 1 Horselethill Road, Glasgow, G12 9LX UK; 3 School of Medicine, University of Dundee, Mackenzie Building, Kirsty Semple Way, Dundee, DD2 4BF UK; 4 Sandyford Sexual Health Services, 2-6 Sandyford Place, Glasgow, G3 7NB UK

**Keywords:** Empathy, Sexual health nurses, Community healthcare, Validation studies, CARE measure

## Abstract

**Background:**

Increasingly healthcare policies emphasise the importance of person-centred, empathic care. Consequently, healthcare professionals are expected to demonstrate the ‘human’ aspects of care in training and in practice. The Consultation and Relational Empathy (CARE) Measure is a patient-rated measure of the interpersonal skills of healthcare practitioners. It has been widely validated for use by healthcare professionals in both primary and secondary care. This paper reports on the validity and reliability of the CARE Measure with sexual health nurses.

**Methods:**

Patient questionnaires were collected for 943 consultations with 20 sexual health nurses. Participating patients self-completed the questionnaire immediately after the encounter with the nurse. The questionnaire included the ten item CARE Measure, the Patient Enablement Index, and overall satisfaction instruments. Construct validity was assessed through Spearman’s correlation and principal component analysis. Internal consistence was assessed through Cronbach’s alpha and the inter-rater reliability through Generalisability Theory. Data were collected in 2013 in Scotland.

**Results:**

Female patients completed 68% of the questionnaires. The mean patient age was 28.8 years (standard deviation 9.8 years). Two of the 20 participating nurses withdrew from the study. Most patients (71.7%) regarded the CARE Measure items as very important to their consultation and the number of ‘not applicable’ and missing responses’ were low (2.6% and 0.1% respectively). The participating nurses had high CARE Measure scores; out of a maximum possible score of 50, the overall mean CARE measure score was 47.8 (standard deviation 4.4). The scores were moderately correlated with patient enablement (rho = 0.232, *p* = 0.001) and overall satisfaction (rho = 0.377, *p* = 0.001. Cronbach’s alpha showed the measure’s high internal consistency (Cronbach’s alpha coefficient = 0.95), but the inter-rater reliability could not be calculated due to the high achieved CARE Measure scores that varied little between nurses.

**Conclusions:**

Within this clinical context the CARE Measure has high perceived relevance and face validity. The findings support construct validity and some evidence of reliability. The high CARE Measure scores may have been due to sample bias. A future study which ensures a representative sample of patients on a larger group of nurses is required to determine whether the measure can discriminate between nurses.

## Background

Patients consistently regard the human qualities of their health care as a top priority [1–3]. Empathy is central to the patient-practitioner relationship [4] and has been linked to higher patient satisfaction [5, 6], patient enablement and improved health outcomes [7–12]. Empathy appears to be a crucially important issue for patients across different countries and cultures [13, 14]. In the United Kingdom (UK), the Francis Report (2003) [15], which details the findings of the public enquiry on the failings of the Mid Staffordshire NHS Trust in England, highlighted that lack of empathic, person-centred care contributed to neglect, suffering and even death of patients. Not surprisingly, the importance of person-centred, empathic care is reflected in healthcare policies [16–18], and in professional bodies [19, 20]. For example, the Nursing & Midwifery Council of the UK [20] states that nurses should ‘treat people with kindness, respect and compassion’, and the Royal College of Nursing highlights the need for an empathic approach in their Principles of Nursing Practice [21]. Around the world, healthcare practitioners are more and more expected to demonstrate the ‘human’ aspects of care in training and in practice. Therefore, obtaining feedback through validated measures is important.

The Consultation and Relational Empathy (CARE) Measure is a patient-rated measure of the interpersonal skills of healthcare practitioners [2, 22] that has been widely validated for use by healthcare professionals in both primary and secondary care settings [22–26]. It is widely used in the UK (including in general practitioners’ appraisal and revalidation) and internationally in various languages [27–31]. Patients complete ten items on the perceived healthcare practitioner’s ‘relational empathy’, which is defined as the practitioner’s ability to:

- “understand the patient’s situation, perspective and feelings (and their attached meanings);
- communicate that understanding and check its accuracy, and
- act on that understanding with the patient in a helpful (therapeutic) way” [2, page S11].

The development of the CARE Measure included reviewing relevant measures and in-depth interviews with patients living in deprived and affluent areas. It was subsequently validated with general practitioners (GPs) in the UK [22]. As person centredness has been promoted within nursing models of care and associated professional nursing practice [32–34] the CARE Measure seems well suited to nursing. However, research on its validation with nurses is limited to one study on routine consultations with practice nurses in general practice [23]. Practice nurses in the UK mainly perform routine annual reviews of patients with different chronic diseases, and some also do minor illness clinics. Wider research with nurses, including those with specialist roles is lacking. Sexual health is an especially sensitive topic and nurses working in sexual health require empathic communications skills [35]. However, there are no specific measures for use in this setting.

## Methods

### Aim and research questions

The overall aim of the current study was to determine the validity and reliability of the CARE Measure as a tool for assessing patients’ views on the quality of healthcare encounters with sexual health nurses working in drop in clinics that provide a range of integrated specialist genito-urinary medicine and reproductive health services [36]. Nurses providing care in ‘drop in’ services are less likely to have serial consultations with patients and hence have limited opportunity to establish empathic relationships with them over time. The specific research questions addressed in the study were:

1. Is the CARE Measure a valid and reliable measure of the quality of the healthcare encounter in routine practice of sexual health nurses?
2. How do the results compare with previous validation studies on different professional groups, and particularly with practice nurses who are more likely to have serial encounters with patients?

### Measures

This was a cross-sectional questionnaire study. The study setting was the public specialist genito-urinary medicine and reproductive drop-in clinics of one Scottish Health Board. All 70 nurses working in the service were invited to participate. The nurses were introduced to the study during a staff meeting that the researchers (AB and BF) attended and an email about the study was sent to all nurses working in the clinics. Recruitment stopped when 20 nurses (from eight clinics) volunteered to participate. All participating nurses gave written, informed consent. The intention was to collect 50 patient questionnaires for each nurse. This number was based on previous studies in doctors that showed that 40–50 questionnaires were required to give a reliable estimate of mean practitioner score [24]. As it was not always possible to determine beforehand whether a patient would consult a nurse who was participating in the study, the receptionists gave questionnaires to all patients who reported at reception. Participating nurses were then asked to write their study identification number on the questionnaire at the start of each consultation so that the questionnaire could be linked to the nurse.

The study questionnaire was similar to the ones used previously. A description of the questionnaire including the CARE Measure is shown in Table 1.

**Table 1:** The study questionnaire

Measure/ item	Description
The CARE Measure.	The 10 items of the CARE Measure are rated on a 5-item response scale from 1 = poor to 5 = excellent. The overall score is the sum of the ten items with 10 being the lowest possible score and 50 the highest. Up to two not applicable (N/A) responses or missing values are allowed and these are replaced by the average item score [22].
Importance of the CARE Measure question	The importance of the CARE Measure items to their consultation was assessed on a 4 point rating scale (from 1 = not important to 4 = very important).
Overall satisfaction question	Overall satisfaction was rated on a Likert scale (from 1 = completely satisfied to 7 = completely dissatisfied). This item was included to obtain evidence of convergent validity. Perceived empathy is known to be an important factor for patient satisfaction. Therefore, the prediction would be that it correlates positively with CARE measure scores [5, 6, 42].
The Patient Enablement Instrument (PEI)	The 6 items contained in the PEI were included to assess divergent validity. Enablement is related to satisfaction and CARE measure scores, but it is a different concept. The predication is that PEI would correlate less strongly with the CARE measure than patient satisfaction [43].
Relational continuity questions	How well the patients knows the nurse was rated on a Likert scale (from 1 = don’t know at all to 5 = know very well). Whether or not previously seen by nurse, consultation length, satisfaction with consultation length was rated from 1 = very poor to 6 = excellent.
Socio-demographic questions	These included self-perceived overall health, age, gender, living arrangements, employment status and language spoken at home.
Questions on waiting time, and satisfaction with waiting time	These two additional questions (from 1 = very poor to 6 = excellent) were included after discussion with the nurses. As a ‘drop in’ service and waiting times can vary considerably it was important to be able to assess the impact of waiting time on the CARE Measure scores.

Patient participation in the study was voluntary and the questionnaire did not elicit any identifiable information about patients or any information about their medical conditions. As in previous studies the patients were asked to fill in the questionnaire immediately after their consultation with the nurse and place it in a sealed box in the waiting area. Data collection was between April 2013 and October 2013. In 2013 the number of visits across all the clinics of the service was 110,300 made by 60,800 individuals (of which 70% female).

### Statistical analysis

Through descriptive statistics the data were summarised, the CARE Measure and Patient Enablement Instrument (PEI) scores calculated, and the variability in the data assessed. Differences between groups were assessed through non-parametric tests, because the data distributions were skewed. To test whether the CARE Measure measured relational empathy in the way that the Measure was designed to measure this concept we assessed its validity in sexual health nurse consultations in several ways. The perceived relevance and face validity [37] (i.e. whether the patients perceived the CARE Measure to measure empathy) of the CARE Measure were examined by calculating the number of not applicable and missing values for each of the ten CARE Measure items, and the patients’ rating of the importance of the ten items. Construct validity [37] (meaning the extent to which the CARE Measure predictably corresponds or not corresponds with other constructs) was assessed through factor analysis (principal component analysis with varimax rotation and Kaiser normalisation and correlations (Spearman’ rho) between the CARE Measure items, and PEI (divergent validity, meaning expect no relation between the constructs) and the overall satisfaction measure (convergent validity, meaning expect the constructs to relate). To assess that the CARE Measure can be interpreted consistently with sexual health nurse consultations we assessed the reliability of the CARE Measure. Internal reliability of the CARE Measure was examined by using Cronbach’s alpa that calculates the inter-correlations of the 10 CARE Measure items. Finally, the inter-rater (patient) reliability of the CARE Measure in providing stable levels of relational empathy to sexual health nurses was assessed by Generalisability-theory (G-Theory). The software packages SPSS (version 21) and urGENOVA via its associated wrapper program GS4 [38, 39] were used to analyse the data as was done in our previous studies [25, 26].

## Results

Completed questionnaires were obtained for 943 consultations. Two nurses who worked part time withdrew from the study because they were unable to collect the required number of questionnaires. Out of the remaining 18 nurses, 15 nurses collected 50 questionnaires or more. It was not possible to identify how many patients refused to participate or were not invited to do so by the reception staff. Female patients completed 637 (68%) of the total number of questionnaires. The patient ages ranged from 16 to 85 years (mean 28.8 years, standard deviation 9.8 years) and the majority of the patients (85%) scored their own health as good or very good. One third of patients (33%) lived with a partner or spouse and almost three fifths of patients (58%) were employed (Table 2).

**Table 2:** Patients’ characteristics

	Sample size (n)	% of total sample
Gender		
Male	288	30.5
Female	637	67.6
Missing values	18	1.9
Age group		
16–29 years	592	62.8
30–44 years	249	26.4
45–65 years	68	7.2
> 65 years	7	0.7
Missing values	27	2.9
Overall Health Status		
Very good/ good	804	85.3
Fair	88	9.3
Bad/ very bad	31	3.2
Missing values	20	2.1
Living arrangements		
With Partner/Spouse	308	32.7
Not with Partner/Spouse	585	62.0
Missing Values	50	5.3
Language Spoken at Home		
English	887	94.1
Other	37	3.9
Missing Values	19	2.0
Employment status		
Employed (full- or part-time, including self-employed)	602	61.8
Unemployed (looking for work)	69	7.1
Unfit to work	45	4.6
In education	197	20.2
Other	44	4.5
Missing	48	4.9
Help with Questionnaire		
Yes	12	1.3
No	889	94.3
Missing Values	42	4.5

Almost four fifths of patients (79%) reported that they had not seen the nurse before. The length of consultations ranged from 1 to 125 min (mean 17.9 min, standard deviation 11.2 min) and the waiting time from 0 to 300 min (mean 47.3 min, standard deviation 46.7 min). More than 9 out of 10 patients (93%) rated the consultation length as very good or excellent, and three fifths of the patients (60%) rated the waiting time as good or higher.

The overall mean CARE Measure score for all participating sexual health nurses was 47.8 with standard deviation 4.4. The scores were not normally distributed (skew −2.4, kurtosis 6.5). The mean CARE Measures scores per nurse ranged from 47.1 to 49.1. There was a moderate range of scores from individual patients from 20 to 50 and 65% of consultations were given the maximum possible score of 50 by participating patients.

CARE Measures correlated weakly/moderately, but positively with consultation length (Spearman’s rho 0.072, *p* = 0.22), satisfaction with consultation length (Spearman’s rho 0.285, *p* = 0.001) and satisfaction with waiting time (Spearman’s rho 0.161, p = 0.001). CARE Measure scores did not correlate with how well patients knew the nurse or with waiting time. Also, the CARE Measure scores did not relate to the patients’ age, gender, self-perceived overall health, living arrangements, employment status and language spoken at home.

### The perceived relevance and face validity

In total, there were 238 ‘not applicable’ and 9 missing item responses, representing 2.6% and 0.1% respectively of the total number of responses for all 10 items. The higher number of ‘not applicable’ responses was mostly explained by two items which asked patients to rate how [good] the nurse was at ‘*helping you to take control*’ (item 9) and ‘*making a plan of action with you’* (item 10) (Table 3).

**Table 3:** Applicability and missing values by CARE Measure items

CARE Measure item	Not Applicable responses (%)	Missing values (%)
item 1 Making you feel at ease	0 (0)	0 (0)
item 2 Letting you tell your story	43 (4.6)	1 (0.1)
item 3 Really listening	8 (0.8)	1 (0.1)
item 4 Being interested in you as a whole person	10 (1.1)	1 (0.1)
item 5 Fully understand your concerns	26 (2.8)	0 (0)
item 6 Showing care and compassion	9 (1.0)	1 (0.1)
item 7 Being positive	7 (0.7)	1 (0.1)
item 8 Explain things clearly	4 (0.4)	0 (0)
item 9 Helping you to take control	58 (6.2)	1 (0.1)
item 10 Making a plan of action with you	73 (7.7)	3 (0.3)
Total	238 (2.6)	9 (0.1)

Almost three-quarters (72%) reported that the CARE Measure items were very important to the consultation. The perceived relevance of the Measure increased with age (*p* = 0.020) (Table 4). No significant association was found in perceived relevance of the CARE Measures scores in relation to the other patient characteristics.

**Table 4:** Patients’ perceived importance of the CARE Measure items to their consultation

	Little or No Importance (%)	Moderate Importance (%)	Very Important (%)	*p*- value
All Consultations	31 (3.3)	194 (20.6)	676 (71.7)	
Age group				0.020
≤ 29	21 (3.7)	136 (24.1)	408 (72.2)	
30–44	8 (3.3)	38 (15.8)	194 (80.8)	
> 45	0 (0.0)	12 (16.0)	63 (84.0)	
Gender				ns
Male	6 (2.2)	62 (22.3)	210 (75.5)	
Female	24 (3.9)	129 (21.1)	458 (75.0)	
Overall Health Status				ns
Very good/good	27 (3.5)	171 (22.1)	576 (74.4)	
Fair	3 (3.6)	11 (13.3)	69 (83.1)	
Bad/very bad	0 (0.0)	8 (26.7)	22 (73.3)	
Living arrangements				ns
With Partner/Spouse	8 (2.7)	63 (21.4)	224 (75.9)	
Not with Partner/Spouse	20 (3.5)	123 (21.7)	423 (74.7)	
Language Spoken at Home				ns
English	29 (3.4)	183 (21.4%)	642 (75.2%)	
Other	1 (2.9%)	8 (22.9%)	26 (74.3%)	
Employment Status				ns
Employed	19 (3.3)	117 (20.1)	445 (76.6)	
Not employed	8 (2.8)	71 (25.3)	202 (71.9)	
Help with Questionnaire				
Yes	0	3 (30.0%)	7 (70.0%)	ns
No	28 (3.3%)	186 (21.7%)	645 (75.1%)	

### Construct validity of the CARE measure in sexual health nurses

Factor analysis on the 10 CARE Measure items, 6 PEI items and 3 satisfaction measures (overall satisfaction, satisfaction with waiting time, and satisfaction with consultation time) identified three factors (Table 5). The CARE Measure items loaded on one factor with high loadings (0.804 to 0.891), suggesting that the Measure has a robust internal structure. The PEI items loaded on the second factor, and the satisfaction measures on the third factor. The three factors explained 73.2% of the variance.

**Table 5:** Factor analysis of the CARE Measure, PEI, and satisfaction items

	Factor 1	Factor 2	Factor 3
1.Making you feel at ease	0.804	0.097	0.172
2.Letting you tell your story	0.829	0.083	0.137
3.Really listening	0.828	0.089	0.109
4.Being interested in you as a whole person	0.838	0.087	0.155
5.Fully understand your concerns	0.891	0.097	0.078
6. Showing care and compassion	0.883	0.104	0.127
7.Being positive	0.860	0.128	0.169
8.Explain things clearly	0.832	0.041	0.083
9.Helping you to take control	0.823	0.115	0.082
10.Making a plan of action with you	0.814	0.107	0.070
PEI 1 Ability to cope with life	0.062	0.825	0.119
PEI 2 Ability to understand illness	0.126	0.864	0.073
PEI 3 Ability to cope with illness	0.112	0.906	0.059
PEI 4 Ability to keep self health	0.090	0.896	0.073
PEI 5 Confidence about health	0.129	0.859	0.098
PEI 6 Ability to help self	0.090	0.895	0.103
Rating waiting time	0.097	0.028	0.826
Rating consultation time	0.176	0.142	0.756
Overall satisfaction	0.281	0.224	0.666

The CARE Measure correlated moderately with overall satisfaction 0.377, *p* = 0.001, thereby supporting convergent validity. As anticipated the CARE Measure correlated less with (divergent) patient enablement (Spearman’s rho 0.232, *p =* 0.001).

### The reliability of the CARE measure with sexual health nurses

The Cronbach’s alpha coefficient of 0.95 indicated high internal reliability of the CARE Measure. Nurses achieved very high CARE Measure scores that varied little between nurses (range of CARE Measure mean scores between individual nurses is 47.1–49.1). The very high scores and the consequent limitation of range impacted on the other forms of reliability analyses beyond that of internal consistency. For example, the analysis of inter-rater (i.e. inter-patient) reliability is dependent upon variance in nurse scores for its numerator. Given a limited range of mean individual question scores for the participating nurses it was not possible to show reliability dependent on differences between nurses. As a result, the inter-rater reliability could not be established in the current study.

## Discussion

Patients who completed the CARE measure were satisfied with their consultations with sexual health nurses. This was reflected in the high scores on the CARE Measure items, which were largely perceived by patients to be highly relevant to their consultations with sexual health nurses. Besides the perceived importance of the items, the low instances of not applicable and missing responses also supported the face validity of the CARE Measure. The CARE Measure inventory indicated high internal reliability (coefficient 0.95), which showed that the CARE Measure items performed well. The CARE Measure items loaded highly on a single factor, which indicates that the items measure the same concept, thereby supporting its construct validity.

The correlation with overall satisfaction was higher than the correlation with enablement and this would tend to support convergent and divergent validity. The high CARE Measure scores for each participating nurse meant that the range of mean CARE Measures scores was limited. As a result the inter-patient reliability was nearly non existent and no differentiation between the nurses could be made based on relational empathy.

A number of factors could have contributed to the consistent high CARE Measure scores for the sexual health nurses. A key consideration is the fact that we were not able to determine how many patients refused to participate or were invited to do so by the reception staff. Thus we cannot assume that the participating patients are representative of all patients attending the clinics. Secondly, unlike general practice nurse consultations, the focus of treatment in the consultations in this study were more likely to be better defined both in terms of patient’s expectation and the practitioner’s response e.g. discussion relating to testing and treatment for sexually transmitted infection or contraceptive choices. It is also possible, that patients in the present study were more likely to feel some anxiety and/or embarrassment in the period leading up to the consultation, so that the high scores post consultation may have reflected the relief of having dealt with the problem. Allied to both these possible reasons, the high scores could also reflect that this group of health practitioners is particularly skilled in demonstrating relational empathy during their patient consultations through training and experience. Finally, the service offers a walk-in system without predefined appointment times. The consultation length is less rigid and is determined by the complexity of the patient’s presenting problem. This could mean that the nurse is able to address the patient’s problem fully and give her/his full attention.

### In comparison with previous studies

As anticipated, the number of patients who had seen the sexual health nurse previously (17%) was much lower than those in a similar study with practice nurses (76%) who are more likely to have consultations with the same patients. However, the mean CARE Measure scores for all sexual health nurses was slightly higher (47.8) than in a similar study with practice nurses (45.9) [23]. It was also higher than the CARE Measure scores with GPs (40.9) [24].

Face validity was similar to that found in previous studies, with high level of perceived relevance of the CARE Measure items to practice nurse consultations (73%) and GP consultations (76%), and low numbers of not applicable responses in both studies. Studies outside general practice [25, 26] and in international settings reported similar findings [27, 28]. That older patients (45 years or more) were more likely than younger patients to score the CARE Measure items as important was also found in previous studies [24] as well as that item 9 (helping you to take control) and item 10 (making a plan of action with you) had the highest instances of ‘not applicable’ responses [24–26, 28, 30]. A possible reason for this could be that most participants rated themselves as healthy and that within the context of their visit as healthy individuals they did not perceive that they needed help to take control or that they did not need further follow up or intervention.

In terms of construct validity, the current study found lower correlations between the CARE Measure, PEI and overall satisfaction than in previous studies [7, 22, 23, 26]. The lower correlations are likely to be due to the limited variation in the CARE Measure scores in this sample. Moreover, in the current study, the CARE Measure could not differentiate between the individual nurses, while in a previous study with practice nurses the inter-rater reliability indicated that 60 patient questionnaires per practice nurse provided a stable basis to evaluate the quality of the encounter for educational and quality improvement purposes [23]. The estimated number of completed CARE Measure was lower (50 CARE Measure questionnaires) in the study with GPs because there was more variability in the data [24].

This means that less patients are needed in order to differentiate between the GPs on relational empathy. As mentioned before, in the current study there was hardly any variability in the CARE Measure data. Therefore, it was not possible within this sample to calculate the required number of patient questionnaires on which feedback to the individual sexual health nurse practitioner should be based.

Previous studies have consistently demonstrated a strong association between continuity of care and high CARE Measure scores. In the present study, sexual health nurses who are less likely to have serial encounters with patients than other practitioner populations studied achieved higher CARE Measure scores. For the reasons previously discussed, it is possible that continuity of care is not important if the purpose of the consultation is very focused for both patient and practitioner.

### Strengths and limitations

A benefit was that the study followed on from previous validity and reliability work on the CARE Measure [23–26], thereby expanding the scope of the CARE Measure across different healthcare professions and settings. Limitations of the study related to the participating nurses being volunteers, and the participating nurses being asked to write their study identification number on the questionnaire at the start of each consultation. As discussed above, we were not able to ascertain how many patients refused to participate or were excluded by the reception staff and this may have introduced sample bias [37]. Another limitation was that only 15 out of the 20 nurses collected the required 50 questionnaires and this could have affected the inter-rater reliability as more questionnaires would have increased the possibility to be able to differentiate between the individual nurses [24].

## Conclusions

Although the CARE Measure could not differentiate between the sexual health nurses in this sample, the face validity, high internal consistency, and construct validity show that the CARE Measure can provide an opportunity for the patients to feedback their experience of the quality of the encounter. As far as we are aware this is the first study in which the calculation of inter-rater (in CARE studies raters are patients) reliability of the CARE Measure was prevented by a lack of variance in subject scores. However, a future study which ensures a representative sample of patients and with a larger group of nurses may be able to show this. Also, further work is required in a similar nursing setting that share some, but not all of the characteristics with sexual health nurse consultations, such as specialist nursing clinics. Doing so will give further insight into the reliability of the CARE Measure to measure the performance of nurse practitioners.

## Abbreviations

CARE Measure: Consultation and Relational Empathy Measure
GP: General practitioner
G-Theory: Generalisability-theory
NMC Code: Nursing & midwifery council code
PEI: Patient enablement instrument
